# Lay beliefs of COVID-19 vaccine refusal among intercity commercial drivers in the Volta region of Ghana: recommendations for improved vaccine uptake

**DOI:** 10.1186/s40794-023-00214-9

**Published:** 2024-03-01

**Authors:** Emmanuel Manu, Mbuyiselo Douglas, Mawuli Komla Kushitor, Joyce Komesuor, Mary Akua Ampomah, Nicholas Obuobisa Opoku

**Affiliations:** 1 Department of Population and Behavioural Sciences, Fred N. Binka School of Public Health, University of Health and Allied Sciences, Hohoe, Ghana; 2 Department of Public Health, Faculty of Health Sciences, Walter Sisulu University, Private Bag X1, Mthatha, 5117 South Africa; 3 Department of Health Policy Planning and Management, Fred N. Binka School of Public Health, University of Health and Allied Sciences, Hohoe, Ghana; 4 Department of Family and Community Health, Fred N. Binka School of Public Health, University of Health and Allied Sciences, Hohoe, Ghana; 5 Department of Epidemiology and Biostatistics, Fred N. Binka School of Public Health, University of Health and Allied Sciences, Hohoe, Ghana

**Keywords:** COVID-19, COVID-19 vaccine, Vaccine hesitancy, Lay beliefs, Ghana, Volta region

## Abstract

**Background:**

The COVID-19 vaccine has faced increased hesitancy in Ghana and the Volta region in particular since its rollout. Acceptance of the vaccine among intercity commercial drivers is crucial, especially in the Volta region, as they transport people within and outside the country and could fuel the transmission of the virus if not vaccinated.

**Objective:**

We therefore established lay beliefs surrounding COVID-19 vaccine refusal among intercity commercial drivers in the Volta region of Ghana, as well as their recommendations for improved vaccine uptake.

**Methods:**

We purposively interviewed twenty-five (25) intercity commercial drivers who had not been vaccinated for COVID-19 in the Volta region of Ghana using a semi-structured interview guide and analysed their responses thematically using the ATLAS.ti software.

**Results:**

Various (ten) beliefs surrounding COVID-19 vaccine refusal were identified. These include the nonexistence of COVID-19, being immune to COVID-19, and the belief in the nonexistence of vaccines and vaccines being meant for the sick. Other beliefs identified were the belief that the COVID-19 vaccine is meant to reduce Africa’s population, that the vaccine triggers other health complications leading to death, the belief that vaccination could cause financial loss, political mistrust, that the COVID-19 vaccine is not permitted by God, and the belief that prayer prevents COVID-19 infection. They also suggested that the adoption of persuasive communication techniques, the publication of information on those who died of COVID-19, providing evidence of tests conducted on the vaccine, testing people before vaccination, provision of care to those who may experience side effects from the vaccine, and being able to explain why varied vaccines are used for the same virus could help improve vaccine uptake.

**Conclusion:**

Our findings show that there is a general lack of understanding and mistrust surrounding the COVID-19 vaccine among intercity commercial drivers in the Volta region. Hence, health promotion officers and communicators in the region need to be knowledgeable on the vaccine as well as on the conspiracy theories thwarting its uptake to provide comprehensive education to the public and intercity commercial drivers to improve its uptake.

## Background

The outbreak of coronavirus disease in 2019 (COVID-19) plagued several countries globally, including Ghana [1, 2]. COVID-19 not only increased the plight of individuals with underlying chronic health conditions [3] but also decimated social and economic normalcy [4, 5]. Public health systems and healthcare infrastructure also came under severe strain globally and on the African continent [6, 7]. Hence, frantic efforts were made by the World Health Organisation (WHO) and various governments to find a lasting antidote to the spread of the pandemic, taking into consideration the ever-increasing cost of personal protective equipment (PPE), such as nose masks and sanitisers [8, 9]. Moreover, the cost of PPE was taking a toll on the finances of individuals, and PPEs were at the same time uncomfortable to use for some people [10]. Furthermore, these measures were temporary; hence, a lasting solution was needed to address the global spread of the pandemic. Hence, the discovery of the COVID-19 vaccine was welcoming to the health community, as it was deemed to halt the spread of the disease upon uptake [11].

The vaccine has faced much hesitancy on the African continent, including Ghana [12, 13]. While appreciable projections were made in Ghana concerning the acceptance and uptake of the vaccine prior to its introduction, such has not been the case since the vaccine was rolled out. Alhasan and colleagues [14] postulated that over 60% (60%) of the Ghanaian adult population were ready to receive the vaccine upon its introduction. Similar projections were made by other researchers [15, 16]. Meanwhile, as of June 2023, only 40.6% of the adult population in the Volta region has been fully vaccinated for COVID-19, with the region being the least vaccinated out of the sixteen regions in the country [17]. This is against the backdrop that the region is the fourth highest in terms of COVID-19 infections in the country, with a case count of 6 187, per data from the Ghana Health Service [18].

Various reasons, such as concerns with vaccine safety, side effects, lack of trust in pharmaceutical industries, and conflicting information from the media, have been reported for COVID-19 vaccine hesitancy on the African continent [19]. Although these reasons have been identified as potential drivers of COVID-19 vaccine hesitancy, they are not exhaustive enough to inform the development of risk communication interventions on the continent in relation to the disease, as factors for vaccine hesitancy could be context- and population-specific [20, 21].

Meanwhile, intercity commercial drivers play a vital role in linking people within the regions and other parts of the country as well as with neighbouring countries such as the Republic of Togo. Hence, the uptake of the COVID-19 vaccine by intercity commercial drivers is essential in curtailing the spread of the disease, as they are likely to encounter individuals who have contracted the disease or have been exposed to it. Therefore, with the low uptake rate of the vaccine in Volta, ascertaining lay causal theories that might hinder the uptake of the vaccine among a key population, such as intercity commercial drivers, is key in understanding the trajectory that COVID-19 vaccination campaigns need to follow to maximise the uptake of the vaccine in the region.

Moreover, although various studies have explored the COVID-19 vaccine acceptance and uptake rates among the Ghanaian populace [12, 14, 16, 22–24], the focus has not been on identifying lay causation theories that influence vaccine refusal. Some of the studies that have investigated COVID-19 vaccine hesitancy in the country have thus far been quantitative in nature [12, 13, 25–27], failing to unearth the “voices” and concerns of key populations such as intercity commercial drivers. Hence, a new tangent on how to maximise COVID-19 vaccine uptake through understanding key populations’ beliefs and perceptions of the vaccine, especially in poorly receptive settings such as the Volta region, needs to be explored. Therefore, we aimed to ascertain the lay causal theories that influence COVID-19 vaccine refusal among intercity commercial drivers in the Volta region of Ghana, following the qualitative reporting guidelines of O’Brien et al. [28].

## Materials and methods

### Study site and period

The study was carried out in the Volta region of Ghana from July to October 2022. The Volta region is located at 3° 45’ latitude N and 8° 45’ longitude N in the eastern corridor of Ghana and covers a landmass of 205,572 km^2^. It stretches from the coast of the Gulf of Guinea and shares common boundaries with four (4) major regions of Ghana, namely, the Greater Accra, Eastern, Bono East, and Oti regions. It consists of eighteen (18) administrative districts, with Ho as the regional capital. The region has an estimated population of 1 659 040 [29]. The region shares a boundary with the Republic of Togo; hence, there are shared commercial interactions between residents of the region and citizens from the Republic of Togo. The region was chosen for the study because it is the region with the fewest vaccinated people (40.6%) for COVID-19 in the country [17].

### Study design

We employed an exploratory qualitative design approach to ascertain the lay theories behind COVID-19 vaccine refusal among intercity commercial drivers in the Volta region. Polit and Beck [30] postulate that the approach is designed to illuminate how a phenomenon is manifested and is especially useful in uncovering the full nature of a little-understood phenomenon. Hence, we adopted the approach to uncover participants’ “hidden beliefs” regarding COVID-19 vaccine refusal.

### Study participants and recruitment process

This study was part of a larger study that was carried out to ascertain the uptake and lay beliefs surrounding the COVID-19 vaccine among intercity commercial drivers in the Volta region of Ghana. Selected participants who indicated not having been vaccinated in the quantitative phase of the study were interviewed on why they had not been vaccinated. Thus, the purposive sampling technique was followed to recruit participants for interviewing, as we had to obtain participants who could provide the information required to address the aims of the study [31]. To be included in the study, participants had to be at least eighteen (18) years of age, cover a driving distance of at least 100 km, and not be vaccinated for COVID-19. Twenty-five (25) participants out of thirty-five (35) people who met the inclusion criteria were interviewed, as the remaining ten dropped out as a result of either being about to embark on their various trips or declining to participate in the study.

### Data collection procedure

A semi-structured interview guide was used to collect the data. The guide was divided into three broad thematic areas: sociodemographic characteristics of the participants’ lay beliefs surrounding the refusal of the vaccine as well as their recommendations for improved uptake of the vaccine. The questions were developed in the English language and translated into Ewe. The guide was piloted among four long-distance commercial drivers from the Oti region which has similar socio-cultural characteristics as the Volta region to help identify and rectify ambiguous questions for clarity.

Depending on the language preference of the participants, interviews were either conducted in English or in Ewe. The interviews were conducted in secluded locations at various lorry stations. Each participant was assigned a numeric code before the interview to not divulge their personal identities during the interview and data analysis processes. The codes also enabled us to link the interviews with the source they emanated from. With the permission of the participants, the interviews were recorded with an Olympus audio recorder. The average duration for the interviews was 45 min. The interviews were conducted by four trained data collectors under the supervision of the principal investigator.

### Data analysis

The interviews were transcribed by an experienced translator in the English and Ewe languages. The interviews conducted in Ewe were transcribed into English and then handed to the Principal Investigator. The transcripts were then duplicated and shared between two independent coders. The transcripts were loaded into ATLAS.ti version 9.0 software for analysis. The analysis was grounded in the thematic analysis approach propounded by Braun & Clarke [32]. Thus, we identified, analysed, and interpreted patterns of meaning, otherwise known as themes from the transcripts, by following the inductive coding process whereby the themes emanated from the data itself rather than being predetermined by the researchers. The coders, together with the principal investigator, immersed themselves in the data by reading and rereading the transcripts, noting similar and unique codes in the process.

After systematically generating initial independent codes from all the transcripts, the codes were collated into potential themes. A three-member theme, comprising the Principal Investigator and the two independent coders, then convened a meeting, reviewed the codes, and further refined them. Areas of disagreement were then resolved by agreeing on the most appropriate phrase (code) in such instances. Final themes were generated by merging similar codes. Unique codes or leftovers were treated as independent themes. Sample compelling extracts were then chosen to demonstrate the nature and scope of each theme in presenting the results. Table 1 presents a summary table of the thematic results generated from the analysis.

**Table 1:** Summary of the thematic table of the study findings

Major theme (Main)	Sub-theme	Code	Frequency
**Lay theories of COVID-19 refusal**			
**Beliefs concerning the COVID-19 vaccine**			
		Non-existence of COVID-19	17
		Immunity	6
		Non-existence of vaccines	4
		Vaccines meant for the sick	5
		Meant to reduce Africa’s population	16
		Triggers other health complications	11
		Causes financial loss	11
		Political mistrust	14
		COVID-19 vaccine not permitted by God	3
		Prayer averts COVID-19	6
**Proposed recommendations for vaccine uptake**			
		Persuasive communication	11
		Publication of information on those who died of COVID-19	6
		Providing evidence on tests conducted on the COVID-19 vaccine	9
		Testing before vaccination	6
		Provision of care to those that experience side effects	8
		Explaining why varied vaccines are used	5

### Rigor

We introduced ourselves to potential participants such that they did not feel intimidated to sincerely respond to the questions. After transcribing the recordings, we contacted some of the participants that we could easily reach to confirm that our transcriptions were true reflections of what they said during the interviews before we proceeded to analyse the data. We also employed two experienced qualitative researchers who were not part of the study to authenticate the transcripts per the recordings by comparing the transcribed documents with the audio recordings to confirm that transcription had been done verbatim before data analysis commenced. With our detailed methodological description, independent researchers should be able to replicate the study should the need be. We also eschew preconceived ideas during the interviews and in the analysis process to minimise researcher bias.

## Results

### Participants’ background information

**Table 2:** Socio-demographic characteristics of participants

Variable	Frequency	Percentage (%)
**Sex**		
Male	25	100
Female	0	0.0
**Age category**		
21–30 years	5	20.0
31–40 years	12	48.0
41–50 years	3	12.0
Above 50 years	5	20.0
**Marital status**		
Married	19	76.0
Single	6	24.0
**Highest educational attainment**		
No formal education	0	0.0
Primary level	5	20.0
Secondary level	18	72.0
Tertiary	2	8.0
**Religious affiliation**		
Christianity	22	88.0
Islam	3	12.0
**Years of driving experience**		
Less than 5 years	4	16.0
5–10 years	5	20.0
More than 10 years	16	64.0

From Table 2, a total of twenty-five (25) interviews were conducted. All participants (100%) were males, with the majority (48%) aged thirty-one (31) to forty (40). The majority (76%) were married and 72% were secondary school graduates. Most (88%) were Christians, and the majority (64%) had more than ten (10) years of driving experience.

### Lay beliefs of COVID-19 vaccine refusal

Participants had varied (ten) beliefs as to why they had refused to receive the COVID-19 vaccine. These include the nonexistence of COVID-19, being immune to COVID-19, and the belief in the nonexistence of vaccines and vaccines being meant for the sick. Other beliefs identified were the belief that the COVID-19 vaccine is meant to reduce Africa’s population, that the vaccine triggers other health complications leading to death, the belief that vaccination could cause financial loss, political mistrust, that the COVID-19 vaccine is not permitted by God, and the belief that prayer prevents COVID-19 infection.

#### Nonexistence of COVID-19

One popular view that most of the participants held was disbelief in the actual existence of COVID-19. They believed the masses were being deceived by the government and the scientific world for the parochial interest by cashing out an imaginary illness called COVID-19. A total of seventeen (17) participants held this view. The following quotes summarise their views:

*There is nothing like COVID-19. I do not believe in it. Were we not in this country when they [the government] told us about a disease called Ebola that was coming? What happened to it? Anytime they [government officials] want to steal money from us [the country], then they come and lie to us about a fake disease. I won’t get vaccinated today or tomorrow. They should allow me to die in peace if there is a disease like that. I am tired [of life] already* [Male, 40 years old].

My brother, there is nothing like that [COVID-19]. Okay, you tell me, have you seen anybody in your family or community that has contracted this disease? We only hear about it on the television and the radio and that should tell you that it is fake. When you talk of illnesses like malaria, I will believe you because we all get it [infected] but for this one [COVID-19], I think the Whiteman just want to lie to us [Africans] and make us use our money to buy their medicines [vaccine] to make more money out of us. I will not contribute to making them [Western world] rich, so I am not going to get vaccinated [Male, 32 years old].

#### Being immune to COVID-19

Another misconception that some participants had about COVID-19 was the belief that they were naturally immune to COVID-19. Six of the participants explained that they either do not easily get sick or have divine protection for COVID-19 and hence would not bother to be vaccinated. Some even explained that getting vaccinated would rather make them contract the illness, as they would have undermined their divine protection and thus be punished for that by contracting the disease. The following quotes summarise their views.

For me, I do not easily get sick. Whenever there is a disease [outbreak] like this, everybody will get it [infected] but I will not. I can’t even remember the last time I went to the hospital for medical reasons. I am fit like a bull, so there is no need for me to get vaccinated. I will survive this like any other disease in the past, Insha Allah [Male, 36 years old].

The God I serve would not allow this disease [COVID-19] to attack me. Do you go to church at all? Don’t you believe in your God? When you have faith in God, things like this [disease outbreaks] do not bother you. As children of God, we are supernaturally immune to COVID-19. My pastor even said that he saw us being vaccinated in the spiritual realm against the disease so there is no need to get vaccinated again [Male, 44 years old].

#### Belief in the nonexistence of vaccines

A section of the participants (four of them) was also of the opinion that vaccines do not actually exist. They referred to the HIV virus about the lack of a vaccine for it. Thus, if vaccines truly existed, people would not get sick in the first place, and scientists would have come out with a vaccine for HIV. They, therefore, believed that the whole COVID-19 vaccine business was a scam. Here is what they had to say:

*For me, I do not believe that vaccines exist. You tell me, how come there is no vaccine for HIV that came long ago but there is a vaccine for COVID-19 which just broke out? If vaccines existed like they want us to believe, by now, every disease will have its own vaccine and we won’t be sick again. That should tell you that there is nothing like that [vaccine]. They are just lying to us to make their money* [Male, 38 years old].

*These people [pharmaceuticals] are liars. Ever since I was a child, cholera has been killing people, but there is no vaccine for that. My grandfather told me that when you offend the gods, that’s when illnesses like these happen. All that we need to do is to pacify the gods for the sickness to go away, not what they are telling us [vaccination]. They don’t work* [Male, 35 years old].

#### Vaccines are meant for the sick

Five of the participants also felt that once they were not sick, there was no need to be vaccinated. To them, vaccines are meant for sick and not healthy people. They believed vaccination weakens the body if you are not sick. Hence, healthy people need not get vaccinated until they fall ill. Here are summaries of what they had to say:

*Why should I be vaccinated when I’m fit and healthy? Medications and vaccines are for the sick, not the healthy. Even the bible says that it is the sick that needs the doctor, not the healthy one. When I get sick because of the disease (COVID-19), then I will think of getting vaccinated* [Male, 44 years old].

*I do not have the time to waste to go and get vaccinated when I know that I am not sick. It is only sick people who go to the hospital. …Even that, if they bring the vaccines here, I won’t vaccinate because I am not sick as I told you. They [health authorities] should first focus on those that are ill* [Male, 29 years old].

#### The belief that the COVID-19 vaccine is meant to reduce Africa’s population

Most of the participants (16) believed that the COVID-19 vaccine is meant to reduce Africa’s population, as it causes impotence and infertility among men. Hence, they vouched never to get vaccinated. They mentioned that there have been several machinations by the West in the past to reduce Africa’s population, and the COVID-19 vaccine is the current tool to achieve that aim. Some of them explained:

*Let me ask you this [question]. Do you believe in this vaccine? Over the past years, these people [the Western world] have been finding ways and means to reduce our [Africa’s] population but have not been succeeding because God is on our side. That is the same agenda they want to implement with this vaccine. If you think I am lying, ask those who have been vaccinated and see how they perform in bed. People’s wives have started divorcing them because they have become impotent because of this vaccine* [Male, 47 years old].

*I have been told that the vaccine causes sexual weakness among men, so why should I go for it? It is another form of family planning they are introducing since their initial plans have failed. I will never be vaccinated* [Male, 39 years old].

#### The vaccine triggers other health complications

Eleven of the participants also refused to get vaccinated because they had either seen or heard that the uptake of the vaccine leads to other health complications and even in some cases, deaths. They claimed that some recipients of the vaccine became diabetic upon vaccination, while others collapsed and died. The fear of these conditions prevented them from getting vaccinated. They explained:

*I know a man in my area who was very healthy, but after getting vaccinated, he started feeling unwell until he was taken to the hospital. He was then told he has diabetes, and he now regrets going for the vaccine. If he had stayed away from it, nothing would have happened to him. I won’t make that mistake* [Male, 27 years old].

I am told that a man in the Western region collapsed and died a few days after taking the vaccine, so I don’t know what will happen to me if I should take it. It won’t risk it [Male, 32 years old].

#### Vaccination causes financial loss

Some participants (11) were also of the view that getting vaccinated would incapacitate them for a while and hence would not be able to go about their daily driving activities, leading to financial loss. They, therefore, refused to be vaccinated as they were the breadwinners of their families. Their views are summarised below:

*Some drivers had headaches for approximately one week after getting vaccinated and were unable to go to work during that period. If it had happened to me, I don’t know how my family was going to survive as I am the sole* breadwinner [Male, 48 years old].

*The time for me to go for the vaccine is not even there. Okay, who will drive my car for me if I should go join a cue to get vaccinated? Even if there is no cue, what if I get sick after vaccination and cannot drive again? Once they say there are side effects, it means that anything can happen and as a family man, it is too much of a risk to take* [Male, 52 years old].

#### Political mistrust

Another key driver of COVID-19 vaccine refusal was political mistrust. Most of the participants (14) were wary about the intentions of the government towards the people in the region as the region is not a stronghold of the government in power. Hence, with the government championing the COVID-19 vaccination campaign drive, participants were hesitant to accept the vaccine. They explained:

*With this government, you must look downward whenever they ask you to look up. Whatever they say is a lie, so I do not believe in this vaccine. I am sure they have something to gain from it that is why they are forcing us to get vaccinated* [Male, 35 years old].

*What at all is making the government so interested in the Volta region [concerning the COVID-19 vaccine]? Are we the only people that have refused to be vaccinated? Why don’t they concentrate on the other regions like they are doing here? Maybe they have a plan to make sure that they reduce our votes in the next elections that is why they are so concerned about us. But for me, whatever they do, I will not get vaccinated* [Male, 44 years old].

#### COVID-19 vaccine not permitted by God

Some of the participants (3) believed the COVID-19 vaccine was not permitted by God and thus was satanic. They saw it as a mark of the antichrist, and true Christians were thus not permitted to be vaccinated. They explained:

*This thing is in the Bible. In the end days, they will try to force all of us to accept the mark of the beast [Antichrist], which I am ready to fight with all my might. I will never get vaccinated even if they make it compulsory. They are just trying to make life difficult for us [Christians] on this earth, but I don’t care* [Male, 35 years old].

*Their [the government’s] diabolic plans against Christians will not work. They want to send all of us to hell because they are already condemned through their corrupt ways, so they want us to accept the mark of the beast [Antichrist] and join them in hell. When they were enjoying their corrupt money, did they share it with me? Why then do they want me to accept the mark of the beast [get vaccinated]?* [Male, 44 years old]

#### Prayer averts COVID-19 infection

Some participants (6) who were Christians who did not deem the COVID-19 vaccine as a mark of the antichrist somehow believed that prayers could protect them or even cure them from a COVID-19 infection. They thus did not see the need to get vaccinated. Their summarised views are presented below:

*We declared a whole month’s fasting and prayer against the disease (COVID-19) so as a man of faith, I know that what God cannot do does not exist, so I won’t waste my time to go and get vaccinated. I know the God I serve; He has never disappointed me* [Male, 36 years old].

*I am an elder in my church who leads prayers and encourages the congregation to believe in the power of prayer, so what signal will I be sent to the congregation if I am seen to fear this virus? Is the disease mightier than the God we serve? With prayer, all things are possible* [Male, 49 years old*].*

### Proposed recommendations for improved vaccine uptake

Despite participants’ hesitation to be vaccinated against COVID-19, they suggested some ways through which uptake of the vaccine could be improved among commercial drivers in the Volta region. They suggested that the adoption of persuasive communication techniques, the publication of information on those who died of COVID-19, providing evidence of tests conducted on the vaccine, testing people before vaccination, provision of care to those who may experience side effects from the vaccine, and being able to explain why varied vaccines are used for the same virus could help improve vaccine uptake.

#### Adoption of persuasive communication techniques

Some participants (11) felt that they were being scared through health communication messages to get vaccinated instead of persuading them. They were of the belief that if health communication messages implored messages that were persuasive enough, it could improve the uptake of the vaccine rather than trying to scare them.

Here is a summary of what they had to say:

*I think they should try to convince people nicely to get vaccinated instead of trying to scare them. If you tell me that I will die if I don’t get vaccinated, it won’t force me to get vaccinated because everyone will die someday. They should find something to tell us* [Male, 47 years old].

*Some of the health workers are rude. When they approach you and you say you will not take the vaccine, instead of them talking nicely to you, they become rude. Nice talk always wins people over so they should try to talk nice to us* [Male, 38 years old].

#### Publication of information on those who died of COVID-19

Most of the participants (16) were also of the opinion that if data on COVID-19 mortality rates, including the identities of the deceased, were made known, it could have made people believe in the existence of COVID-19 and thus made them take the vaccine. They said:

If they truly want us to get vaccinated, they should let us know those that have died of the disease. We are always told that a certain number of people have died from COVID-19, but no one knows them. I have been in this town for a long time, and we hear people have died from the disease, but we don’t know them [Male, 40 years old].

They should come clear on those who have died from this disease. Apart from former President Rawlings, whom it was said died from COVID-19, I do not know anyone that it has killed again. Therefore, we think they are just lying to us. If indeed they want me to get vaccinated, I need proof that the disease exists [Male, 32 years old].

#### Providing evidence of tests conducted on the vaccine

Some participants (9) also suggested that providing evidence on the processes leading to the development of the vaccine could boost public confidence in its uptake of the vaccine. They indicated that the hast through which the vaccine was developed gives credence to the impotence hypothesis that people had about the vaccine. They explained:

*Looking at how quickly the vaccine was developed; I think they should come clean and let us know how it was produced. Even HIV has been with us for all this while how come there is no vaccine for it but for COVID-19? This makes us believe that they have different agendas. However, if they can come and explain things [the processes] better, some of us might understand them [health authorities] and get vaccinated* [Male, 35 years old].

*If they truly want us to get vaccinated, they should be ready to tell us the truth about the vaccine. They should come with proof that the vaccine won’t give us problems like infertility if we take it. Who tested it [the vaccine] in Ghana to prove that it is good for us in this country? They should come and tell us* [Male, 26 years old].

#### Testing people before vaccination

Some participants (6) were also of the opinion that people should be tested first and that those found to be infected should be vaccinated instead of mass vaccination. In that way, people will be motivated to take the vaccine since they have already contracted the vaccine. Two of them explained:

*I think the only way is to test the people before giving them the vaccine. If I know that I have the disease, why won’t I vaccinate? However, if I’m healthy as I am and you don’t test for me to know whether I have the disease or not, then I won’t vaccinate. It is as simple as that* [Male, 36 years old].

*Like I said earlier, those that need the vaccine the most are those that are sick [infected]. Therefore, they should test everybody and then vaccinate all those who are sick. In that case, you can’t say you won’t take it [get vaccinated], and the government will also save money. That is my opinion* [Male, 50 years old].

#### Provision of care to those who may experience side effects

Eight of the participants were also of the opinion that a proper healthcare provision system to address COVID-19 vaccination-related side effects could encourage individuals to accept the vaccine. They lamented that the after-care services that are provided to mitigate side effects are inadequate, thus discouraging them from going for the vaccine. Two of them explained:

They are forcing people to get vaccinated, but they don’t care about those that fall sick after getting vaccinated. I knew a man who fell ill after taking the vaccine, and when he went to the hospital, they said his insurance had expired. Therefore, how do you convince others to get vaccinated? [Male, 46 years old]

They only tell you to take paracetamol in case you experience headaches or body pains but don’t give you the paracetamol themselves. Some people may need more than paracetamol after the injection, so who will take care of those ones? I think these are some of the things they should improve to encourage people to get vaccinated [Male, 33 years old].

#### Explaining why varied vaccines are used for the same virus

One of the key aspects of COVID-19 vaccine education that needs to be considered is explaining how different vaccines are used for the same virus, as it will go a long way to improve its uptake. Five participants were confused as to why more than one vaccine variant was used to combat the disease, giving credence to various conspiracy theories. Two of the participants explained:

*They [health authorities] also need to explain to us why there is more than one vaccine type for the same virus. Is there something they are hiding from us? I think making things clear [on why the various vaccine types] could encourage people to get vaccinated* [Male, 38 years old].

*When malaria came, it was quinine only that was used to treat it for a very long time until other medicines came later. However, with this disease [COVID-19], there are so many of them [vaccines] already so it appears to be everything was planned. However, if they can explain why we have different vaccines for the same disease, maybe I will get vaccinated* [Male, 45 years old].

## Discussion

### Lay beliefs of COVID-19 vaccine refusal

In this study, we ascertained the lay beliefs that influence COVID-19 vaccine refusal among intercity commercial drivers in the Volta region of Ghana, as their uptake or refusal of the vaccine could promote or minimise the transmission of the virus since they are key players in the transport industry, especially in these regions where the vaccine has been poorly accepted. Various beliefs (ten) were identified to promote COVID-19 vaccine refusal.

One of the reasons that some participants shun the COVID-19 vaccine was their belief that COVID-19 does not exist. They were convinced that the viral outbreak was a scam created by the superpowers and health authorities to siphon monies. This, therefore, made them hesitant to be vaccinated. Conspiracy theories have surrounded the COVID-19 outbreak right from the onset, as some believed that the virus was deliberately manufactured by the Chinese to wage war on the USA [33]. Similar conspiracy theories were reported by Uscinski and colleagues [34]. Meanwhile, conspiracy theories regarding science, medicine, and health-related topics, if not promptly and effectively dealt with, can prompt people to eschew appropriate health-related behaviours such as the refusal of the COVID-19 vaccine by some intercity commercial drivers in the Volta region of Ghana [35]. It could therefore be argued that appropriate education on the disease might not have been carried out by health authorities in the regions and the country at large to dispel the disbelief in the existence of the illness, which needs to be addressed if wider acceptance of the COVID-19 vaccine is to be achieved.

Another belief that made people refuse the vaccine was the belief that they were naturally immune to the virus. Although without any scientific evidence, this category of participants felt they were naturally fit in a way that they could not be easily infected by the virus, as they had not fallen ill for quite a long time. Although mucosal surfaces combat certain respiratory infections of viral origins using both unspecific and specific mechanisms, such as the production of virus-induced cytokines and natural killer cell activity, as they are the first portals of entry for most infectious diseases [36, 37], this had not been ascertained in the case of our study participants. They thus relied on their history of good health to assume natural immunity to COVID-19. In the case of COVID-19, natural immunity has only been reported among those who have recovered from the disease [38]. While **herd immunity** could provide some form of natural immunity, it often happens when most of the population has been vaccinated [39]. Hence, with the Volta region being the region with the lowest percentage of vaccinated people for COVID-19, **herd immunity** could not be achieved at this stage. Thus, education on natural immunity and COVID-19 must be intensified among intercity commercial drivers in the two regions to improve acceptance of the COVID-19 vaccine.

We also found that some participants did not believe in the existence of vaccines. Aside from the fact that some participants did not believe in the existence of COVID-19, some also believed that vaccines do not actually exist. They buttressed their viewpoint with the fact that the human immunodeficiency virus (HIV), alongside diseases such as cholera, has been in existence for a long time. Hence, if vaccines did exist, such diseases should have been eradicated. It has been reported that the core of anti-vaccine beliefs is a conspiracy theory that vaccines do not work [40], as found in this study. The belief that COVID-19 does not exist among some Africans [41] gives credence to the school of thought that vaccines, especially the COVID-19 vaccine, do not exist among our study participants. This calls for intensified education on vaccines as to how they are developed as well as their mode of action to boost public confidence in the COVID-19 vaccine and vaccines in general.

Some of the participants were also of the notion that vaccines were meant for the sick; hence, as they were not yet sick from COVID-19 infection, they saw no need to be vaccinated. This clearly shows participants’ lack of understanding of the role of vaccines in disease prevention. They had a misconception that vaccines are for curative purposes instead of prevention. Thus, they did not understand the difference between medication and vaccination. The lack of understanding of how vaccines work could be linked to the low literacy levels among our study participants, as most of them had no tertiary education. Research has shown that there is a link between one’s level of education and good health literacy [42, 43]. Moreover, the lack of education, be it formal education or sensitisation about vaccines, in general, has affected vaccine uptake on the African continent [44] and could be the case in this study. Hence, there is a need to improve the health literacy levels of intercity commercial drivers in the two regions for them to be able to make informed healthy choices, thereby accepting the COVID-19 vaccine.

Another salient finding was the belief that the COVID-19 vaccine is meant to reduce Africa’s population by rendering men impotent. The conspiracy theory that the Western world, through pharmaceutical companies, wishes to control Africa’s population growth through various medical interventions has lingered on for a long time. Africans have been very skeptical about public health interventions such as vaccinations for a long time. One common rumour is the perception that they would be rendered sterile by toxic compounds given under the guise of improving health, such as through vaccinations [45]. The perception sometimes takes a religious route, as anti-vaccine propaganda in 2011 intensified that vaccine is Western intrigue to sterilise Muslim girls [46]. Moreover, tales about the Tuskegee syphilis study whereby African Americans were allegedly infected with syphilis have led to widespread mistrust of public health interventions among minorities, the poor, and marginalised groups [47]. Thus, our findings reveal a deep-rooted fear and mistrust of Western-engineered vaccine-related interventions, as they often believe such interventions have ulterior motives. Hence, alongside education, pharmaceuticals could strive to locally produce essential vaccines, such as the COVID-19 vaccine, on the African continent by involving local scientists in the production and education processes to alleviate mistrust.

Some participants were also of the belief that the COVID-19 vaccine triggers other health complications leading to untimely death upon vaccination. They claimed to have known people who, upon taking the vaccine, either developed hypertension, diabetes, or rashes. Some even claimed they knew people who died a few days after vaccination and were convinced that such deaths were related to the COVID-19 vaccine. Although some neurological complications, such as fever, chills, headache, fatigue and swelling or pain at the injection site, have been associated with the COVID-19 vaccine [48], these are often mild. However, cardiovascular complications such as myocarditis, immune thrombocytopenia, and cerebral sinus venous thrombosis have been found to adversely affect the health of some vaccinees [49]. Moreover, participants’ fear of death upon vaccination is not also far-fetched, as a mortality rate of at least 8.2 per million population has been associated with COVID-19 vaccination, with individuals with comorbidities such as hypertension, dementia, chronic obstructive pulmonary disease (COPD), diabetes, and heart failure at a higher risk of death [50]. It has thus been suggested that the public, for that matter intercity drivers in the Volta region, be provided with accurate reports on COVID-19 vaccine-related morbidity and mortality from trusted experts to address their concerns about the vaccine [49].

Some participants were also of the belief that the vaccine incapacitates people to the extent that they are unable to undertake their daily economic ventures, leading to financial loss. They were therefore not willing to take such risk, as they were the sole breadwinners for their families. It is true that the reported neurological and cardiovascular effects of COVID-19 vaccination could temporarily incapacitate people and in some cases lead to untimely loss of life [48, 49]. In such instances, individuals could lose some income. Hence, while treating individuals for these side effects to get them healthy for work, some form of financial incentives could be provided for those who may experience the severe form of these side effects to alleviate their financial loss and boost public confidence in the uptake of the vaccine.

Another major belief that was found to thwart the uptake of the COVID-19 vaccine among participants was political mistrust. This group of participants had an apprehension concerning the government’s intentions in promoting the uptake of the vaccine in the region. Considering the fact that the Volta region is the major stronghold of the leading opposition party in the country made participants wary that the government might have ulterior motives concerning the uptake of the vaccine in the region other than health reasons. Our finding has been corroborated by a similar study conducted in the Oti region of Ghana [51]. Even with essential childhood vaccinations, institutional and political mistrust has been found to decrease the uptake of such vaccines on the African continent [52]. Outside the continent, political mistrust has also been found to thwart vaccine uptake among right-wing politicians in New Zealand [53]. It is therefore prudent that essential vaccination drives in the region and the country such as the COVID-19 campaign drive be led by apolitical individuals and entities, to increase public trust in the process and improve the acceptance and uptake of these vaccines.

Some participants also deemed the COVID-19 vaccine to be a symbol of the antichrist, meaning that it was designed to sway people from God, and as a Christian one was not permitted by God to be vaccinated. To them, getting vaccinated meant accepting the mark of the devil as biblically prophesized; hence, they were bent on not getting vaccinated. A similar perception towards the COVID-19 vaccine has been reported among some Nigerian Christians [54, 55]. This finding shed light on how deep-rooted religious beliefs and sentiments could thwart preventive public health campaigns, which have been found to have serious consequences for preventive medicine [56]. This is because the unvaccinated remain potential carriers and transmitters of the COVID-19 virus. To avert this, it is suggested that religious leaders be empowered to sensitise their followers, including intercity commercial drivers in the Volta region, to the need to get vaccinated against COVID-19.

Although some of the Christian participants did not deem the COVID-19 vaccine to be a symbol of the antichrist, they believed in the power of prayer to avert COVID-19 infection. Even in situations where they would get infected, they believed they would be healed through prayer and hence did not see the need to get vaccinated. Research has shown that belief in the power of prayer could facilitate or hinder adherence to COVID-19 preventive measures, including COVID-19 vaccine uptake [57]. As found in our study, it rather served as a hindrance to vaccine uptake among some participants. Our findings express the limited understanding of the virulence of COVID-19 among the Ghanaian public. However, in situations where proper engagements have been held with communities and faith-based organisations, better outcomes in terms of adherence to prevention measures have been achieved [58].

### Proposed recommendations for improved vaccine uptake

Participants also provided insights into what ought to be done to address the beliefs fuelling COVID-19 vaccine refusal. One such suggestion was the adoption of persuasive communication techniques instead of fearmongering by health communicators to get people to accept being vaccinated. They explained that some health workers and communicators threaten them with death as a consequence of their action (vaccine refusal); however, such a strategy only encourages some people to take entrenched positions against the vaccine. This view has been shared by James and colleagues [59], who argue that persuasive messaging that invokes prosocial vaccination and social image is effective in increasing vaccine acceptability. A similar approach has been suggested by Limaye et al. [60]. This highlights the need for the deployment of professional health promoters and communicators vexed in persuasive communication for COVID-19 vaccine campaigns in the country if desired results are to be achieved.

Another suggestion raised by the participants to boost acceptance of the COVID-19 vaccine was the publication of information on COVID-19 mortality cases. They believed the reported COVID-19-associated deaths were untrue, as they had never seen a known person dying from the disease. Hence, if evidence could be presented for them to believe that some people have indeed died from the disease, it could improve vaccine uptake. Research has shown that when individuals perceive an illness to be severe or susceptible, they are more likely to adhere to its prevention measures, such as vaccine acceptance [61, 62]. Thus, despite the awareness created of COVID-19, some participants in this study did not perceive the disease to be severe, as they had not seen a close relative dying from the disease, hence their refusal to get vaccinated. Although names of COVID-19 victims cannot be published for ethical reasons, in some cases, permission could be sought from family members of the deceased to be used for awareness creation purposes to improve acceptance of the COVID-19 vaccine.

Moreover, participants suggested that providing evidence of tests conducted on the vaccine could improve its acceptance. Due to the hurry in which the vaccine was developed and fueled by the conspiracy theory that it is aimed at depopulating Africans, some people are apprehensive about being vaccinated. Hence, if the processes leading to the development of the vaccine could be thoroughly explained to the public, it could serve as evidence leading to public approval of the vaccine for improved acceptance, as the issue of public trust and confidence in the COVID-19 vaccine and how it impacts its acceptance has already been reported [63, 64].

Another salient suggestion for improved acceptance of the COVID-19 vaccine was the practice of mass testing before vaccination. They believed that people would be convinced to be vaccinated if they tested positive for the virus through mass testing campaigns. Despite the economic implications of this suggestion [65], if deployed, it could boost vaccine uptake in two ways. First, it could be used as a tool to educate people on the need to be protected through vaccination when tested negative and could also furnish health workers with first-hand information on the severity of COVID-19 in local communities to drive uptake. Thus, the perceived benefits of being vaccinated and the perceived severity and susceptibility to the virus could be contextually explained. In addition, this suggestion has been hailed as one of the underexplored strategies for COVID-19 control [66], making participants’ suggestions a legitimate claim.

Effective treatment and provision of compassionate care to individuals who experience side effects from the COVID-19 vaccine was another mechanism participants felt could be deployed to improve the acceptance of the COVID-19 vaccine. The vaccine has been associated with neurological complications such as fever, chills, headache, fatigue, and swelling or pain at the injection site [48] as well as cardiovascular complications such as myocarditis, immune thrombocytopenia, and cerebral sinus venous thrombosis [49]. Since these conditions could be debilitating and immobilise vaccinees for a while prompt treatment and management are required so as not to cause economic or life loss to these individuals. This could boost public confidence in the vaccines and the vaccination process. This is paramount, as the fear of adverse effects of the COVID-19 vaccine has increased apprehension towards it globally [67].

Last, participants mentioned that if health authorities could explain to the public why varied vaccines are being used for the same virus, it could help address some of the misconceptions they have towards the vaccine and thus improve uptake. They could not grasp why and how different vaccines were produced for a singular virus within a short space of time. This heightened their mistrust of the vaccine and increased hesitancy. Various vaccines have been produced to combat the virus, as the various vaccines have varied immunological considerations and strategies owing to their sources of development [68]. However, this understanding is lacking among the general population, including intercity commercial drivers. Hence, educational and awareness campaigns aiming to improve the uptake of the COVID-19 vaccine need to incorporate into their strategies the rationale behind the diverse vaccines. This could again help to boost public confidence and trust in vaccines and improve uptake.

## Conclusion

The findings from this study showed that there is a general lack of understanding and deep-seated mistrust surrounding the COVID-19 vaccine among intercity commercial drivers in the Volta region of Ghana. Hence, health promoters and communicators in the Volta region need to be knowledgeable on the vaccine as well as on the conspiracy theories thwarting its uptake to provide comprehensive education to the public and intercity commercial drivers in particular to improve its uptake.

### Strengths and limitations of the study

Our methods and processes were thorough enough to make our findings credible enough to add to the body of literature on COVID-19 vaccine uptake in Ghana and in similar settings and could also help inform policy on COVID-19 vaccine rollout in the Volta region and the country as a whole. However, although we have kept a proper audit trail of our methods to ensure the credibility of our findings, with only twenty-five (25) participants interviewed, the findings should be generalised with caution. Moreover, only males participated in the study, hence, the socio-cultural beliefs that may affect the uptake of the COVID-19 vaccine among females in the Volta region may not have been captured in this study.

## Data Availability

All data generated or analysed during this study are included in this published article.

## List of abbreviations

COVID-19: Coronavirus Disease 2019
PPE: Personal Protective Equipment
UHAS-REC: University of Health and Allied Sciences Research Ethics Committee
USA: United States of America
